# A Data‐Driven Inverse Design Methodology for Magnetic Soft Millirobots Navigating in Confined Spaces

**DOI:** 10.1002/advs.75714

**Published:** 2026-05-19

**Authors:** Ziyu Ren, Hong Wang, Chak Wang Tse, Yi Zheng, Xi Chen, Wenqi Hu

**Affiliations:** ^1^ School of Mechanical Engineering and Automation Beihang University Beijing China; ^2^ Department of Mechanical and Aerospace Engineering The Hong Kong University of Science and Technology Kowloon Hong Kong China; ^3^ Division of Integrative Systems and Design The Hong Kong University of Science and Technology Kowloon Hong Kong China; ^4^ The Hong Kong University of Science and Technology Kowloon Hong Kong China

**Keywords:** Bayesian optimization, Confined‐space navigation, Cosserat‐rod model, data‐driven inverse design, magnetic soft robotics, sim‐to‐real

## Abstract

Magnetic soft millirobots enable untethered locomotion in narrow environments. However, their design remains largely intuition‐driven due to complex interactions among soft‐body deformation, magnetic actuation, and environmental contact. Here, we propose an uncertainty‐aware, data‐efficient inverse design methodology tailored for contact‐rich, non‐smooth environments. It integrates a physics‐based Cosserat rod model with Gaussian Process‐based Bayesian optimization to automate robot design for confined‐space crawling. To mitigate sim‐to‐real discrepancies, domain randomization is incorporated by explicitly modeling contact uncertainty. Channel segmentation focusing on critical geometric bottlenecks enhances efficiency and accuracy, halving optimization time and increasing R^2^ by nearly an order of magnitude in a serpentine channel. Optimized robots consistently outperform arbitrarily selected baseline designs, achieving stable crawling across heterogeneous conditions without failures such as coiling or jamming. When applied to coronary artery‐mimicking geometries, the optimized design reached 2.66 mm/s, nearly doubling the average baseline speed of 1.42 mm/s. Validation in an artery‐mimicking channel with 2.5 mm out‐of‐plane undulations further demonstrates the reliability of the optimization framework; notably, the optimized design maintained uninterrupted motion while 25% of baseline designs got stuck. This work establishes an uncertainty‐aware inverse design methodology for task‐driven magnetic soft millirobot design, paving the way toward automated design‐to‐deployment pipelines for real‐world applications.

## Graphical abstract

A data‐efficient inverse design framework automates the optimization of magnetic soft millirobots for confined‐space navigation. Integrating a physics‐based Cosserat rod model with Bayesian optimization efficiently identifies high‐performance geometries. Furthermore, frictional domain randomization and geometric segmentation ensure robust sim‐to‐real transfer and computational efficiency, enabling reliable undulatory crawling across heterogeneous 2D and 3D anatomical pathways.

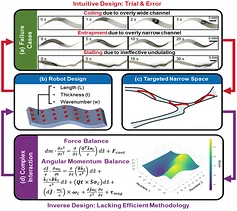

## 1 Introduction

Small‐scale magnetic soft robots can be wirelessly controlled to navigate confined spaces and narrow passages [1]. These robots are typically fabricated from magnetic soft composites, synthesized by embedding hard magnetic microparticles into a polymer matrix [2, 3]. Due to the high remanence of these microparticles, the magnetization distribution of the composite can be programmed, allowing the robots to undergo a wide range of deformations. However, designing these robots presents significant challenges.

**FIGURE 1:**
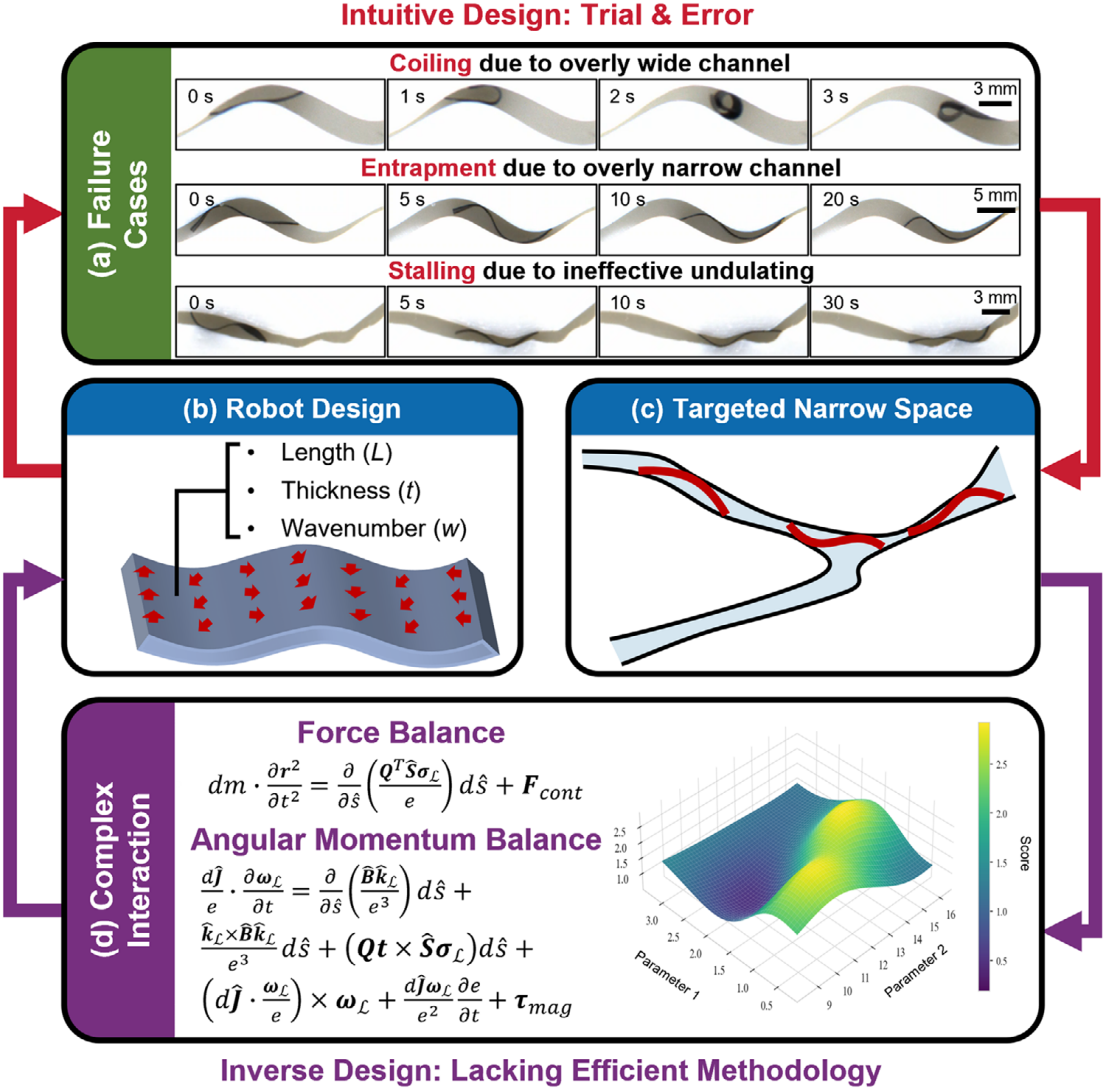
Overview of the scientific challenge. (a) Three typical failure cases observed in channels when robots are intuitively designed. (b) The three design parameters explored in this study: robot length (*L*), thickness (*t*), and wavenumber (*w*). Red arrows represent the magnetization profile. (c) Schematic of the targeted narrow space. (d) The complex interactions occurring when the robot crawls in confined spaces prevent the development of an efficient inverse design methodology.

A prominent example of this design complexity is exemplified by the magnetic soft sheet‐shaped millirobot with a harmonic magnetization profile [4, 5]. This design is noted for its versatility, exhibiting diverse deformation patterns and locomotion modes under varying actuation signals and environmental conditions. In confined spaces, such as narrow channels and slits, these robots can employ an undulatory crawling mode for propulsion, characterized by a traveling wave propagating toward the locomotion direction. In such scenarios, locomotion emerges from the coupled interaction among the magnetically induced torques, the robot's inherently nonlinear magneto‐elastic deformation, and the intermittent contact interactions with the surrounding walls. The interplay among these three factors makes the mapping from design parameters (robot length, *L*, thickness, *t*, and wavenumber, *w*) to locomotion performance highly nonlinear and analytically intractable [5]. As illustrated in Figure 1a, suboptimal designs result in locomotion failures, such as coiling due to overly wide space, entrapment by overly narrow channel, or stalling due to ineffective undulating (Video S1). Predicting and preventing these failures within complex channel geometries remains non‐trivial [6], and existing design paradigms struggle to address these challenges efficiently.

Traditionally, overcoming such design hurdles has relied heavily on human intuition, which often fails to capture complex environmental interactions. To complement human experience, theoretical models have been developed to carry out forward prediction or simulation on robot deformations [2]. These models are either based on constitutive equations tailored for hard‐magnetic soft materials [5, 7, 8, 9] or by distributing torques across discrete elements [10]. While these models allow researchers to validate designs before fabrication, the design process remains a manual “trial‐and‐error” loop, which is inherently inefficient for exploring the vast design space.

To move beyond manual iterations, various inverse design methodologies have been proposed. However, most existing studies primarily focus on static or quasi‐static deformations [11, 12, 13, 14, 15], often simplifying or omitting the complex dynamic interactions between the deformed soft body and its environment due to inherent modelling challenges and substantial computational costs [5, 16]. To address this, simplifications like using Euler–Bernoulli beam theory have been introduced [17], facilitating the inclusion of fluid‐drag terms that account for robot‐fluid interactions [18]. While computationally efficient, these simplified models still struggle to accurately capture the highly non‐linear contact forces and dynamic robot behaviors during actuation in confined spaces.

To bypass the computational burden of physical solvers while maintaining high prediction accuracy, data‐driven surrogates like artificial neural networks (ANNs) have emerged [19, 20, 21]. However, ANNs are data hungry, which can be impractical when high‐quality data collection involves expensive or time‐consuming experiments and simulations. Furthermore, these models can be sensitive to noise, potentially compromising their accuracy and generalizability in real‐world applications [22]. These two challenges are especially true for medical scenarios where the data for each patient is expensive and subject to variations [23, 24].

In contrast to data‐heavy inverse design algorithms, Bayesian optimization (BO) is suited for expensive and noisy optimization problems that involve relatively low‐dimensional design spaces, providing a natural solution to the challenges presented above. Particularly, it bypasses the need for gradient derivation, which is critical for the optimization of sheet‐shaped robot crawling; during the locomotion, the robot may experience abrupt changes in locomotion speed due to environmental transitions (e.g., jamming or coiling), indicating a non‐smooth objective function.

While BO has shown great success in soft robotics, its applications predominantly focus on either control adaptation and gait optimization for fixed morphologies [25, 26, 27, 28], or quasi‐static inverse design without complex dynamic environmental interactions [29, 30]. In fixed‐morphology control tasks, the underlying dynamical system, along with its boundary conditions and contact mechanics, remains invariant. Consequently, locomotion failures are primarily input‐dependent and can often be dynamically reversed on‐the‐fly by adjusting external signals. Furthermore, in existing morphological optimization works, bypassing dynamic contact forces allows the optimization landscape to remain relatively well‐behaved. In contrast, applying BO to soft millirobots’ inverse design in confined, contact‐rich environments goes beyond tuning inputs or static deformations, as it fundamentally alters the underlying dynamical system itself. A slight change in design variables redefines the system's intrinsic mechanics, drastically altering its spatiotemporal deformation and highly non‐linear intermittent contacts. Consequently, poor morphological designs may trigger irreversible failure modes, such as coiling or entrapment (Figure 1a), which cannot be rescued by subsequent control strategies. This dynamic physical coupling renders the optimization landscape extremely challenging.

Motivated by these considerations, we propose a tailored design methodology for the sheet‐shaped magnetic soft millirobot, specifically optimized for navigation in confined environments using BO with GP. To address the bottlenecks of this contact‐rich, non‐smooth optimization landscape, we introduce two strategic methodological integrations. First, a channel segmentation strategy is proposed to evaluate locomotion performance on a set of critical geometric bottlenecks, which effectively simplifies the objective function evaluation and significantly reduces computational time. Second, to bridge the reality gap where sim‐to‐real discrepancies in contact dynamics typically lead to severe locomotion failures, domain randomization [31, 32] is employed. This uncertainty‐aware approach endows the optimized geometries with intrinsic robustness, enabling direct sim‐to‐real transfer without the need for post‐fabrication tuning. Given the known shape of the confined environment, this methodology can automatically generate a robot design to achieve fast crawling speed within a few dozen iterations. We also comprehensively validated the effectiveness of the proposed approach through experiments. While capturing the complexity of the soft‐bodied interaction with the environment, this methodology maintains a good balance between computation cost and data efficiency.

## 2 Overall Optimization Framework

**FIGURE 2:**
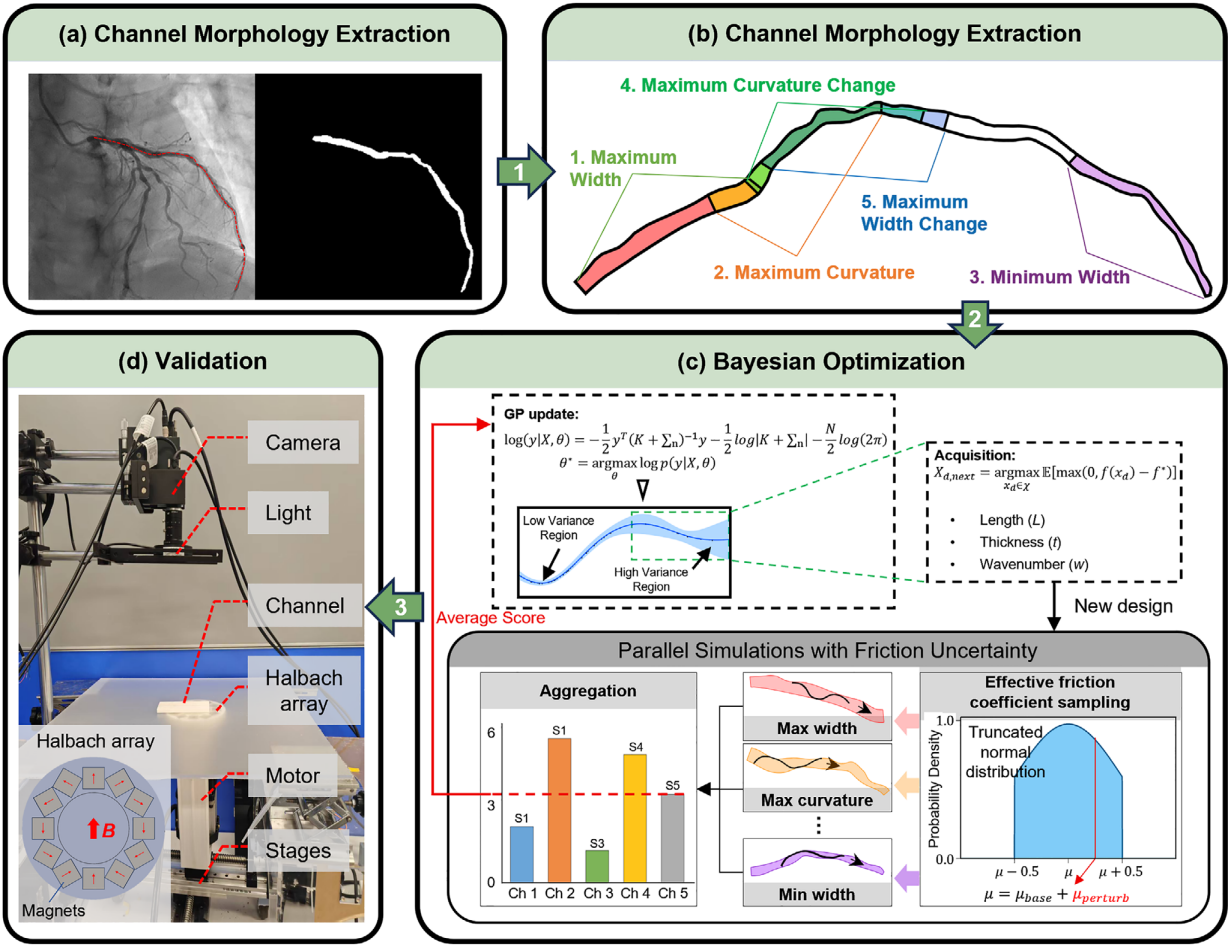
Overall framework of the inverse design methodology. (a) Target environment extracted from an x‐ray angiogram, with the red dashed line indicating the desired vascular channel. (b) Illustration of the five critical sections identified by the segmentation algorithm. (c) Workflow of the Bayesian optimization process utilizing a Gaussian process. (d) Experimental setup for validation tests. The illustration on the lower left shows the Halbach array used to generate a uniform magnetic field.

The overall framework of the inverse design methodology is illustrated in Figure 2. The process begins with the definition of the targeted environment, such as the extraction of the digitized channel geometry from X‐ray angiographic images.

For long or complex channels, we implement a channel segmentation strategy to mitigate the high computational cost of full‐length locomotion simulations. Full‐scale simulation is often redundant because the robot crawling performance within a given channel is limited by the segments with geometric features that are hard to navigate rather than the total path length. We identified five critical geometric features that define the most challenging parts of a channel based on our previous experimental observations [5] (Figure 2b): (1) the maximum width, (2) the maximum curvature, (3) the minimum width, (4) the maximum curvature change, and (5) the maximum width variation. By considering these challenging sections, we can evaluate performance solely within these segments rather than across the entire channel.

Once the simulation environment is established, the optimization algorithm is deployed. As discussed in introduction, the algorithm should be gradient‐free and data‐efficient. These requirements are well‐suited for Bayesian optimization using Gaussian Process. In this framework, the objective functions, specifically, the average locomotion speed for non‐segmented channel cases, and total time spent in each segment for segmented channel cases, are modeled by GP. An acquisition function selects the next set of design parameters with the highest probability of maximizing the average speed or minimizing the transversal time. These candidate parameters are then tested concurrently across the five most challenging channel segments identified. To further account for real‐world uncertainties, effective friction coefficients are randomized in each iteration at every time step. It should be noted that this effective parameter accounts for a broader range of complex macroscopic contact mechanics beyond pure surface friction, encompassing interfacial material adhesion and localized viscoelastic material deformation. The randomization facilitates the discovery of an optimal design that is both robust to environmental variations and efficient in locomotion. The optimization concludes when the iteration count reaches a predefined limit.

Finally, the optimized design is experimentally validated against control groups comprising arbitrarily selected baseline designs. It is worth noting that defining a reasonably optimized intuition‐based design for comparison is challenging. This difficulty arises from the compromises required to balance locomotion reliability and speed across highly coupled design parameters. To eliminate human subjectivity and demonstrate the performance baseline of unguided manual attempts, a set of arbitrarily selected random designs was employed as the control group. An X‐Y planar stage controls the position of a rotating cylindrical Halbach array (Figure 2d), which generates a uniform rotating magnetic field in the validation region. Throughout this paper, we fix the rotating frequency at 4 Hz and the magnetic flux density at 50 mT. The robot navigates within a channel positioned above the cylindrical Halbach array. To maintain the robot within the uniform magnetic field, the X‐Y stage tracks the robot's motion automatically. A camera provides real‐time top‐view feedback of the robot's position to a controller, which actuates the stage to synchronize the Halbach array's movement with the robot.

The subsequent sections provide implementation details for the forward model, effective friction coefficient randomization, and the segmentation strategy.

## 3 Physics‐Based Forward Model

The deformation of the millirobot is modeled based on the Cosserat rod theory [33]. The model accounts for all six degrees of freedom of the filament deformation, allowing bending, twisting, stretching, and shearing. Unlike simpler models based on Euler–Bernoulli theory, the Cosserat model relaxes assumptions such as inextensibility and cross‐section orthogonality, offering a comprehensive and accurate representation of the robot's dynamics [33]. The dynamics of the robot are governed by the balance of linear and angular momenta expressed as:

(1) $$dm\cdot \;\frac{\partial^{2}r}{\partial t^{2}}=\frac{\partial }{\partial \hat{s}}\;\left(\frac{Q^{T}\hat{S}\sigma_{L}}{e}\right)d\hat{s}+F_{cont}$$

(2) $$\begin{array}{cc} \frac{d\hat{J}}{e}\cdot \;\frac{\partial \omega_{L}}{\partial t} & =\frac{\partial }{\partial \hat{s}}\left(\frac{\hat{B}{\hat{k}}_{L}}{e^{3}}\right)d\hat{s}+\frac{{\hat{k}}_{L}\times \hat{B}{\hat{k}}_{L}}{e^{3}}d\hat{s}+\left(\mathrm{Qt}\times \hat{S}\sigma_{L}\right)d\hat{s}\\ & \;+\left(d\hat{J}\cdot \frac{\omega_{L}}{e}\right)\times \omega_{L}+\frac{d\hat{J}\omega_{L}}{e^{2}}\frac{\partial e}{\partial t}+\tau_{mag} \end{array}$$

Here, the central line of the robot is described by $r\left(s,t\right)\in R^{2}$ in the laboratory frame, and the rotation matrix ***Q*** (*s*, *t*) transforms the vector in the laboratory frame to the local material frame (***d***_1_, ***d***_2_, ***d***_3_) defined by the orientation of the cross‐section, where ***d***_3_ aligns with the normal vector of the cross‐section. The Cosserat rod theory allows ***d***_3_ to deviate from the tangent of the central line ***t***, thus incorporating shear and elongation strains. Parameters with subscript L are represented in the material frame. $\sigma_{L}=Q\left(et-d_{3}\right)$ describes the strains associated with shear and axial elongation, and $e=ds/d\hat{s}$ defines the local stretch ratio. $\omega_{L}$ denotes the angular velocity. Parameters with a hat represent the quantities measured in the rest reference frame. $\hat{B}$ and $\hat{S}$ represent the bend/twist stiffness matrix and shear/stretch matrix, respectively. ${\hat{k}}_{L}$ denotes the generalized curvature. $\hat{J}$ is the second moment of inertia of the infinitesimal mass *dm*. ***F**_cont_* and ***τ**_mag_* are contact force and magnetic torque vectors. Equations (1) and (2) represent the balance of linear momentum and the balance of angular momentum, respectively.

The bending rigidity matrix ***B*** and the shear rigidity matrix ***S*** must be provided according to the cross‐sectional geometry. The bending rigidity matrix ***B*** can be expressed as:

(3) $$B=diag\left(EI_{1},EI_{2},GI_{3}\right)$$

where *E* is the Young's modulus of the material with a linear deformation assumption. $G=\frac{E}{2(1+\nu )}$ is the shear modulus with the Poisson's ratio ν = 0.5. Assuming the robot thickness is *t*, the robot width is *b*, the moment of inertia along the robot's transverse direction is given by $I_{1}=\frac{bt^{3}}{12}$, and the moment of inertia along the robot's longitudinal direction $I_{2}=\frac{tb^{3}}{12}$. The polar moment of inertia is approximated as *I*_3_ = *I*_1_ + *I*_2_. The shear rigidity matrix ***S*** can be expressed as:

(4) $$S=diag\left(S_{1},S_{2},S_{3}\right)$$

where *S*_1_ = *S*_2_ = α*JA* represent the shear rigidities along the robot's longitudinal and transverse directions with *A* = *bh* representing the cross‐sectional area. The constant $\alpha =\frac{5+5\nu }{6+5\nu }$ is the shear coefficient for a rectangular cross‐section [34]. *S*_3_ = *EA* denotes the stretch rigidity.

To account for the distributed magnetic torques provided by the external magnetic field, the term $\tau_{L}$ can be written as:

(5) $$\tau_{L}=M_{L}\;\times {\left(\mathrm{QH}\right)}_{L}$$

where $M_{L}$ is the magnetization profile in the material frame, and ***H*** is the magnetic flux density of the applied magnetic field in the laboratory frame. In our study, we assume ***H*** is uniform within the working space and the robot is subjected to pure torques.

The external force ***F**_cont_* comes from the normal and shear forces between the robot and the boundaries. Therefore, ***F**_cont_* = ***F**_n_* + ***F**_f_*. The normal force ***F**_n_* can be expressed as:

(6) $$F_{n}=\left(1-H\left(\varepsilon \right)\right)\cdot \left(-F_{n}^{internal}\cdot n+H\left(\varepsilon \right)k_{w}\varepsilon -\gamma_{w}v\cdot n\right)n$$

where ε is the distance between the robot and the boundary. ε > 0 denotes the robot is away from the boundary, while ε < 0 denotes the robot penetrating the boundary. *H*(ε) is a Heaviside function ensuring the contact force only appears when the robot is in contact or penetrating the boundary. $-F_{n}^{internal}\cdot n$ calculates the robot's internal force along the normal direction ***n*** of the local boundary, where the negative sign denotes that the contact force acts in opposite direction to the internal force. *k_w_* is the contact stiffness coefficient to impose a penalty on penetration. γ_*w*_ is the dissipation coefficient to represent the energy loss in contact. The effective friction ***F**_f_* is modeled as:

(7) $$F_{f}=\left\{\begin{array}{cc} -\max\left(\left|F_{total}\right|,\mu_{s}\left|F_{n}\right|\right)\cdot \frac{F_{total}}{\left|F_{total}\right|} & \;if\;\left|v\right|\leq v_{\in }\\ -\mu_{k}\left|F_{n}\right|\cdot \frac{v}{\left|v\right|} & \;if\;\left|v\right|>v_{\in } \end{array}\right.$$

where ***F**_total_* is the total force acting on the robot. µ_*s*_ and µ_*k*_ are static and kinetic effective friction coefficients, respectively. *v*_ε_ is the velocity threshold to determine whether the robot is slipping or not relative to the boundary.

The simulation environment is implemented based on the open‐source package PyElastica [35], with necessary adaptations as described above. To ensure numerical stability, the Courant‐Friedrichs‐Lewy condition must be satisfied by properly selecting an appropriate time step *dt* given the segment length *dl*. In this work, we adopted *dl_target_* = 0.25 mm. We defined *n_elem_* = *int*(*L_robot_*/*dl_target_*), and added 1 to *n_elem_* if *n_elem_* · *dl_target_* < *L_robot_*. Finally, we computed *dl* = *L_robot_*/*n_elem_* and *dt* = $0.4\cdot \frac{dl}{\sqrt{\left(E/\rho \right)}}$, with *E* at 2.2 MPa representing the robot's Young's modulus and ρ at 1795 kg/m^3^ representing the robot density.

**FIGURE 3:**
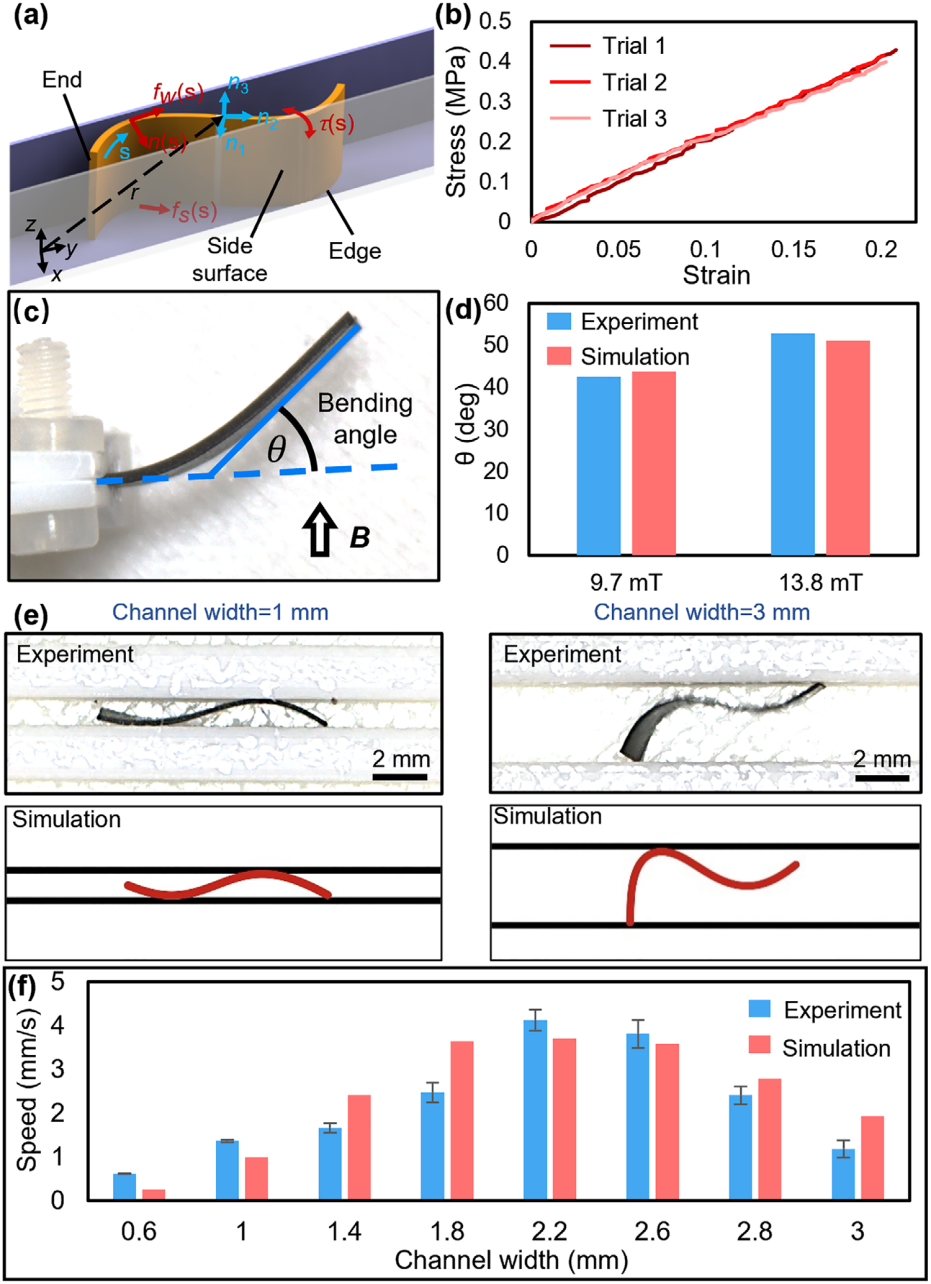
Parameter identification and real‐to‐sim transfer. (a) Physical modeling of the robot crawling in a straight channel. (b) Stress‐strain curves of the magnetic soft composite, with different colors denoting independent trials. (c) Testing setup for magnetization measurement. A cantilevered magnetic beam is clamped at one end and bends upward under a vertical magnetic field. (d) Comparison of beam bending angles between experiments and simulations. (e) Snapshots of the robot crawling in 1‐mm and 3‐mm wide channels during experimental and simulated tests. (f) Comparison of robot speeds between experiments and simulations across various channel widths (robot size: *L*, 8.9 mm, *b*, 3 mm, *t*, 0.18 mm, *w*, 1). The experimental results exhibit strong agreement with the numerical simulations (Pearson correlation coefficient r = 0.878, p < 0.01), validating the reliability of the proposed simulation. Another validation set of the simulation can be found in Figure S2 (robot size: *L*, 9.0 mm, *b*, 3 mm, *t*, 0.102 mm, *w*, 1). The error bar represents the standard variation (n = 5 for simulation and experiments).

The Young's modulus of the magnetic soft material, which was the soft composite made by mixing PDMS and NdFeB microparticles with a weight ratio of 1:1, was determined via tensile testing on a universal testing machine (WD, Ailigu Corp), which yielded the stress‐strain curves. We assumed a linear elastic model for simplicity and calculated the modulus over a strain range of 0% to 5%, obtaining a modulus of 2.2 ± 0.11 MPa (Figure 3b).

The magnetization of the composite was identified through an inverse fitting approach. We measured the bending angles of a cantilevered magnetic beam with known geometry under various magnetic field strengths (Figure 3c). SciPy least squares function was then employed to identify the magnetization value that minimized the discrepancy between the simulated bending angles and the experimental measurements as shown in Figure 3d.

Effective friction coefficients between the robot and the channel surfaces were similarly determined through optimization. We distinguished between three contact pairs, assigning static and kinetic coefficients to each: the robot ends against channel walls ($c_{end}^{static}$, $c_{end}^{kinetic}$), the robot side surface against channel walls ($c_{surface}^{static}$, $c_{surface}^{kinetic}$), and the robot edge against the substrate ($c_{edge}^{static}$, $c_{edge}^{kinetic}$). A Bayesian optimization was performed to find the optimal set of these six coefficients with two additional coefficients, *k_modulus_* and *k_mag_* that are multiplied by the Young's modulus and fitted magnetization density to account for remaining discrepancies, by matching the simulated locomotion speeds to experimental data in straight channels of known widths. As shown in Figure 3f, the simulation results generated using the calibrated parameters show strong agreement with the experimental measurements used for this calibration (Pearson correlation coefficient r = 0.878, p < 0.01). The identified parameters are summarized in Table 1. As we mentioned before, friction coefficients here represent effective friction coefficients rather than pure Coulombic friction coefficients. This composite value is intended to encapsulate a broader range of dissipative forces, including friction, interfacial material adhesion, and viscoelastic material deformation, which may not be fully captured by simpler contact models. Consequently, their fitted values may appear larger than what one might expect for a purely frictional coefficient.

**TABLE 1:** Coefficients set fitted via Bayesian optimization.

Coefficient Parameter	Fitted Value
$c_{end}^{static}$	13.25
$c_{end}^{kinetic}$	1.79
$c_{surface}^{static}$	8.94
$c_{surface}^{kinetic}$	3.79
$c_{edge}^{static}$	0.13
$c_{edge}^{kinetic}$	0.02
*k_modulus_*	1.12
*k_mag_*	1.54

To rigorously validate the sim‐to‐real effectiveness of these parameters against a completely independent experimental dataset, a robot of a different size (*L*, 9 mm, width (*b*), 3 mm, *t*, 0.102 mm, and *w*, 1) was fabricated for subsequent experimental testing (Figure S2). Even with this new robot, the simulated average speeds across various channel widths remained in strong agreement with the experimental average speeds (Pearson correlation coefficient r = 0.957, p < 0.001). The significant variation in speeds observed in the 2‐mm and 2.2‐mm channels is attributed to the robots’ self‐curving behavior, which was treated as a failure case with a speed of 0.

## 4 Bayesian Optimization Under Environmental Uncertainty

While deterministic optimization yields designs tailored to specific target channels, such solutions often fail to account for real‐world uncertainties. Empirical observations suggest that even with consistent designs, locomotion speeds can vary significantly within the same channel. Crucially, the design must also be adaptable to uncharacterized environments, as the precise a priori measurement of effective friction coefficients for all potential channels is impractical.

To address these challenges, we randomize the effective friction coefficient during the optimization process. The objective is to identify a robust design that maintains consistent performance across a wide range of environmental conditions, rather than pursuing a theoretical maximum speed for a single specific scenario. This trade‐off is essential, as designs optimized for peak performance may exhibit high sensitivity to environmental variations, leading to rapid performance degradation under off‐nominal conditions. In the simulation, these physical uncertainties are modeled as stochastic fluctuations in the effective friction coefficients between the robot and the channel surface.

We define the objective function as the expected locomotion performance of a design *x_d_* under stochastic environmental conditions. Mathematically, this is expressed as the expectation of the performance metric over the distribution of effective friction coefficients:

(8) $$J\;\left(x_{d}\right)=E_{c∼TN}\;\left[f\left(x_{d},c\right)\right]$$

where, $x_{d}\in \chi$ is a set comprising three continuous parameters: robot length (*L*), robot thickness (*t*), and number of waveforms (*w*), which acts as a wavenumber multiplier (2π*w*) to define the robot's harmonic magnetization profile. These three scalar parameters were chosen because, as demonstrated in our previous work [5], they dictate the dynamic magneto‐elastic deformation and spatial contact mechanics that govern the robot's locomotion speed and reliability in confined spaces. *c* is defined as:

(9) $$c=c_{nom}+\delta$$

where $c_{nom}\in R^{6}$ is defined as:

(10) $$c_{nom}={\left[c_{end}^{static},\;c_{end}^{kinetic},c_{surface}^{static},\;c_{surface}^{kinetic}c_{edge}^{static},\;c_{edge}^{kinetic}\right]}^{T}\;$$

The perturbation vector δ is defined component‐wise. For the *j*‐th component, the perturbation δ_*j*_ is given by:

(11) $$\delta_{j}=0.5\cdot c_{nom,j}\cdot \xi_{j}$$

where ξ_*j*_ represents the normalized stochastic fluctuation, sampled from a truncated normal distribution *TN*(0, 1) within the interval [‐1, 1]. Consequently, the goal of the optimization is to identify the optimal design $x_{d}^{*}$ that maximizes *J*(*x_d_*) within the given design space χ:

(12) $$x_{d}^{*}=\underset{x_{d}\in \chi }{\mathrm{argmax}}J\left(x_{d}\right)\;$$

Since *J*(*x_d_*) cannot be computed analytically, we employ a GP as the surrogate model. The robot's sensitivity to the variations of friction coefficients depends on the robot design *x_d_*. Robust robot designs yield consistent speeds with low variance, whereas sensitive designs exhibit high performance volatility. Consequently, the uncertainty in the estimation of the locomotion performance varies across the design space. To capture this, the GP models the relationship between the true expected locomotion performance *J*(*x_d_*) and the noisy simulation observations as:

(13) $$y\;\left(x_{d,i}\right)=J\left(x_{d,i}\right)+\varepsilon_{i},\varepsilon_{i}∼N\left(0,\sigma_{n,i}^{2}\right)$$

Here, *y*(*x*_*d*,*i*_) is the observed expected locomotion performance for design *x*_*d*,*i*_. The error term ε_*i*_ captures the stochastic noise in the observation, and it is modeled as a normal distribution with zero mean and variance $\sigma_{n,i}^{2}$. $\sigma_{n,i}^{2}$ is the squared standard error of the mean (SEM) derived from the *M* simulation samples.

A Matérn‐2.5 kernel with Automatic Relevance Determination is applied to quantify the similarity between any two design vectors *x_d_* and *x*′_*d*_ in the design space:

(14) $$k\;\left(x_{d},{x^{\prime }}_{d}\right)=\sigma_{s}^{2}\left(1+\sqrt{5}r+\frac{5}{3}r^{2}\right)\exp\left(-\sqrt{5}r\right)$$

where *r* is the weighted Euclidean distance between the design vectors, scaled by the length scale parameters:

(15)

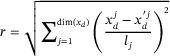




In this equation, $x_{d}^{j}$ refers to the *j*‐th dimension of the design vector *x_d_*. $\sigma_{s}^{2}$ is the signal variance, and *l_j_* are the individual length scale parameters for each dimension. The model updates hyperparameters $\theta =\{\sigma_{s}^{2},l_{1},\text{…},l_{\dim(x_{d})}\}$ at each optimization iteration by maximizing the marginal log‐likelihood (MLL). For heteroscedastic noise, the MLL is defined as:

(16) $$\log\left(y|X,\theta \right)=-\frac{1}{2}y^{T}{\left(K+\Sigma_{n}\right)}^{-1}y-\frac{1}{2}\log\left|K+\Sigma_{n}\right|-\frac{N}{2}\log\left(2\pi \right)$$

(17) $$\theta^{*}=\underset{\theta }{\mathrm{argmax}}\log p\left(y|X,\theta \right)\;$$

where *K* is the kernel covariance matrix computed from all observed data points, *y* is the vector of all observed expected speeds, and $\Sigma_{n}=diag\left(\sigma_{n,1}^{2},\text{…},\sigma_{n,N}^{2}\right)$ is the diagonal matrix of observation noise variances.

The optimization has two phases. First, *N_init_* (specific values are given in respective cases) quasi‐random points are generated using a Sobol sequence to initialize the GP model. Subsequently, at each optimization iteration, a single candidate point is selected by maximizing the q‐Noisy Expected Improvement acquisition function with a batch size of *q* = 1:

(18) $$X_{d,next}=\underset{x_{d}\in \chi }{\mathrm{argmax}}E\left[\max\left(0,J\left(x_{d}\right)-y^{*}\right)\right]\;$$

Here, *X*_*d*,*next*_ denotes the selected candidate design for the next iteration. The baseline y* is the best expected performance identified from the historical data.

Each candidate point is evaluated using Monte Carlo simulation to approximate the intractable expectation. For the chosen design *x*_*d*,*i*_ within a given channel or channel segment at iteration *i*, we conduct *M* = 5 parallel simulations under different friction conditions sampled from the distribution of *c*. The sample mean serves as the observed value *y*(*x*_*d*,*i*_), and the uncertainty in this estimate is quantified by SEM, σ_*n*,*i*_:

(19) $$y\;\left(x_{d,i}\right)=\frac{1}{M}\;\sum_{j=1}^{M}v\left(x_{d,i},c_{j}\right),\sigma_{\left(n,i\right)}=std\left(v_{i}\right)/\sqrt{M}$$

The optimization continues until the desired total number of evaluations is reached.

## 5 Optimization Without Channel Segmentation

**FIGURE 4:**
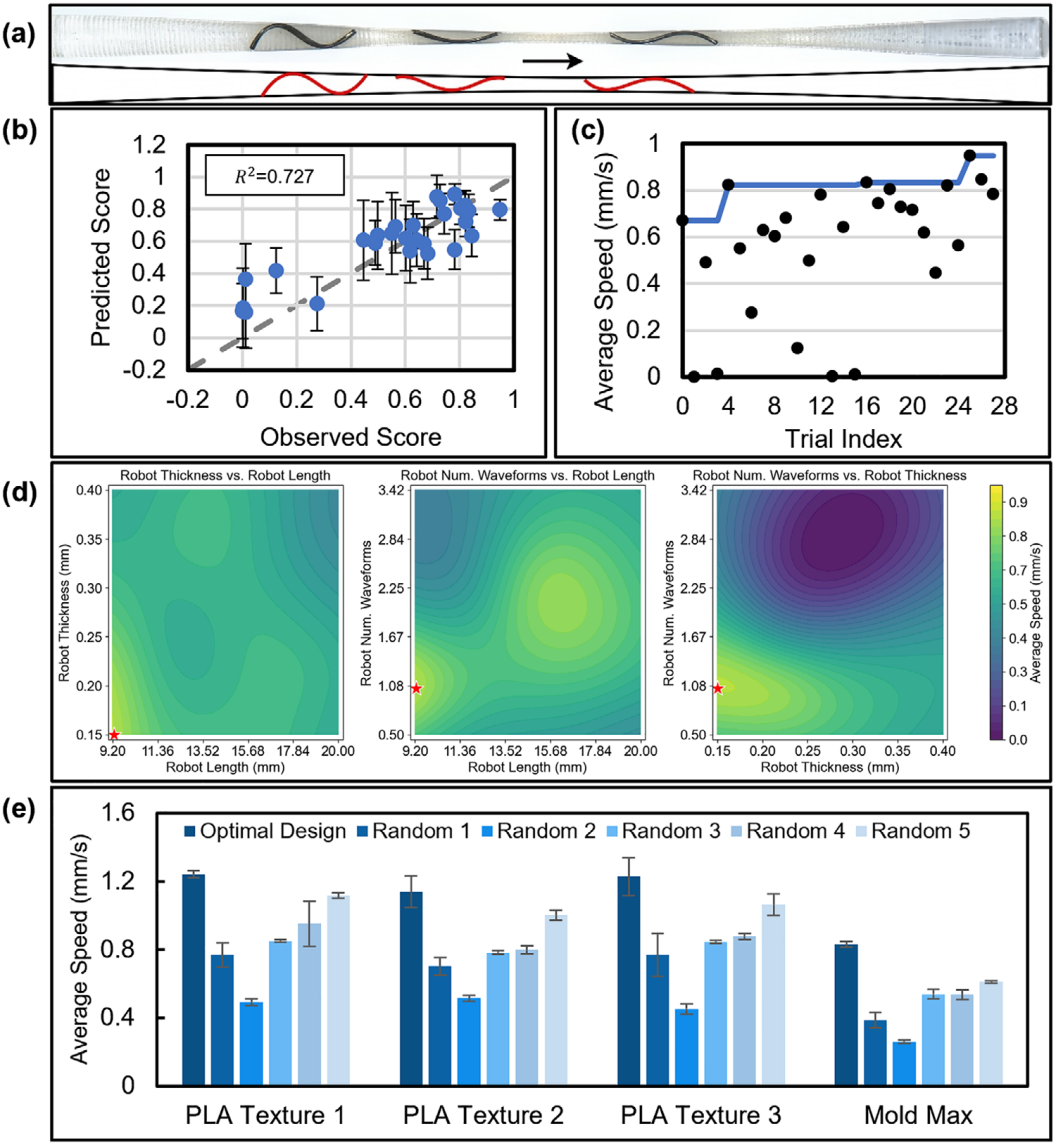
Validation of the proposed Bayesian optimization framework using the non‐segmented strategy. (a) Funnel‐shaped channel used in simulations and experiments. The top row shows experimental snapshots of robot locomotion, while the bottom row displays the corresponding simulation results. (b) Cross‐validation plots for the optimization model. Average speed (mm/s) serves as the score. Vertical bars represent the standard deviation, and the dashed diagonal line indicates a perfect model fit. *R* ^2^ denotes the coefficient of determination. (c) Optimization history from trial 0 to 27. Black dots represent individual trial results. The blue line indicates the maximum average speed achieved. Improvement plateaued at Trial 25 with an average speed of 0.95 mm/s. (d) Optimization landscapes for each design parameter pair. Brighter green regions correspond to higher average speeds. Red stars mark the identified optimal design parameter set. (e) Experimental comparison of robot speeds between the optimal design and randomly selected design parameters (details of random robots’ size can be found in Table S1). The optimal design exhibits the highest speed across various channel textures and materials with different effective friction coefficients. The error bar represents the standard variation (n = 6 total trials across N = 2 independent robots).

As shown in Figure 4, to validate the proposed Bayesian optimization framework, the optimal design was sought for a 2D funnel‐shaped channel characterized by a maximum width of 1.5 mm at the distal ends, tapering to a minimum width of 0.6 mm at the center. Given its simple geometry and relatively short length of 50 mm, simulations were performed across the entire span without segmentation. The optimization aimed to maximize the average locomotion speed over 28 trials, comprising 16 trials generated from a Sobol sequence and 12 trials directed by the acquisition function.

As illustrated in the cross‐validation analysis in Figure 4b, the fitted Gaussian process model achieved an R^2^ of 0.727. This indicates a satisfactory level of predictive accuracy, considering the inherent stochasticity introduced by domain randomization. The found maximum average speed of the robot increased with iterations, demonstrating convergence as depicted in Figure 4c. Upon reaching the predefined limit of 28 iterations, the optimization algorithm converged to a design close to the global optimum predicted by the GP surrogate model (marked by a star in Figure 4d).

The resulting optimal robot parameters were *L* = 9.25 mm, *t* = 0.15 mm, *w* = 1.05. Experimental results conducted across four distinct surface textures, including polylactic acid (PLA) textures 1, 2, 3, and silicone rubber (Mold Max 30, Smooth‐On, Inc.), validated the efficacy of the proposed framework under various friction scenarios. The optimal design consistently outperformed five randomly selected baseline configurations (Video S2), maintaining a superior average speed across all tested environments, notably reaching approximately 1.24 ± 0.02 mm/s on the PLA Texture 1 surface.

## 6 Efficient Simulation with Channel Segmentation

**FIGURE 5:**
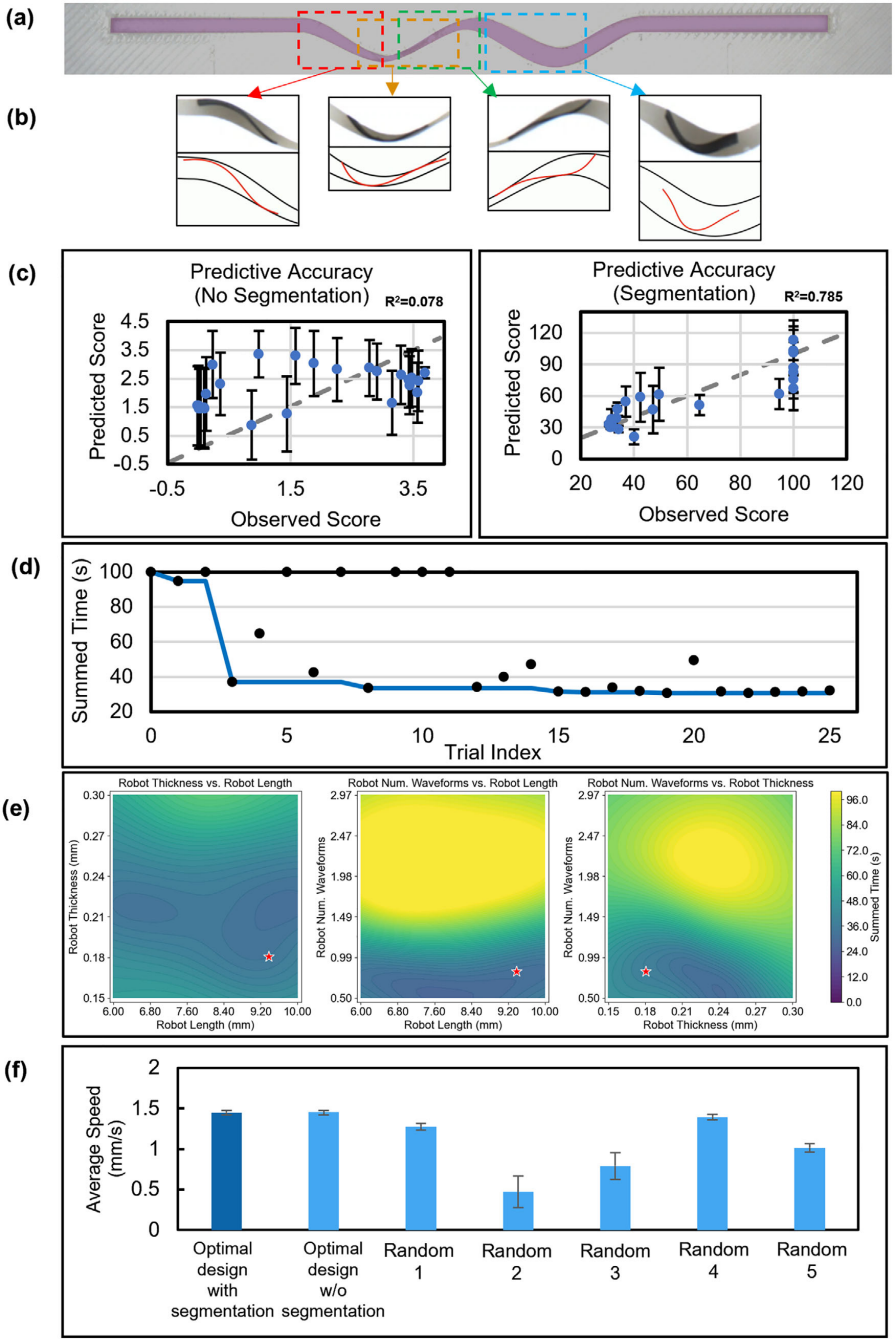
Comparison between the non‐segmented and segmented approaches. (a) The targeted channel used in simulations and experiments. (b) The top row shows experimental snapshots of robot locomotion, while the bottom row displays corresponding simulation results. From left to right, the columns represent segments characterized by: (i) maximum midline curvature change, (ii) maximum midline curvature combined with minimum width, (iii) maximum width change, and (iv) maximum width. Arrows point to the corresponding segments in (a). (c) Cross‐validation plots for the optimization models fitted on the non‐segmented and segmented approaches. Average speed (mm/s) is used as the score in the left plot, while total summed time is the metric in the right plot. Vertical bars represent the uncertainty (standard deviation), and the dashed diagonal line represents a perfect model fit. *R* ^2^ denotes the coefficient of determination. (d) Optimization history from trial 0 to 25 for the segmented approach. Black dots represent trial results, and the blue line indicates the minimum identified total time. Improvement plateaued at Trial 22, reaching a minimum total locomotion time of 30.6 s. (e) Optimization landscapes for each design parameter pair. Darker purple regions correspond to lower total locomotion times. Red stars mark the identified optimal design parameter set. (f) Experimental comparison of robot speeds under the optimal design versus randomly selected designs (details of robots’ size can be found in Table S2). The optimal design demonstrates the fastest speed. The error bar represents the standard variation (n = 6 total trials across N = 2 independent robots).

Optimization efficiency is a critical concern, especially for channels with complex shapes and extended lengths. Furthermore, prolonged full‐channel simulations exacerbate the spatiotemporal accumulation of numerical errors that severely deteriorate surrogate model predictive accuracy. These issues are addressed through a segmentation strategy, which was validated on a 2D serpentine channel whose width varied smoothly between 0.6 mm and 3.0 mm, as shown in Figure 5a. 12 trials were allocated for Sobol sampling, and 14 trials were allocated for subsequent optimization. When full‐channel simulations were used as the optimization objective, the GP surrogate exhibited poor predictive accuracy (R^2^ = 0.078), and each trial required a substantial simulation time (4.4 h). In contrast, segmentation halved the computational cost per trial to 2.2 h while greatly improving the GP model fitting (R^2^ = 0.785), as seen in Figure 5c, enabling more reliable results from the Bayesian optimization routine. The optimal design parameter set obtained without segmentation was *L* = 9.38 mm, *t* = 0.18 mm, *w* = 0.82, while the optimal design derived from segmentation was *L* = 9.00 mm, *t* = 0.22 mm, *w* = 0.79. Both designs were then fabricated and tested experimentally. As shown in Figure 5f, the optimal robot from segmentation exhibited a crawling speed closely matching that of the best full‐channel design and consistently exceeded randomly selected baselines (Video S3). This validates that performance over a small number of carefully chosen segments faithfully represents overall channel navigation. These results establish segmented‐channel optimization as an efficient and robust surrogate for full‐trajectory evaluation.

**FIGURE 6:**
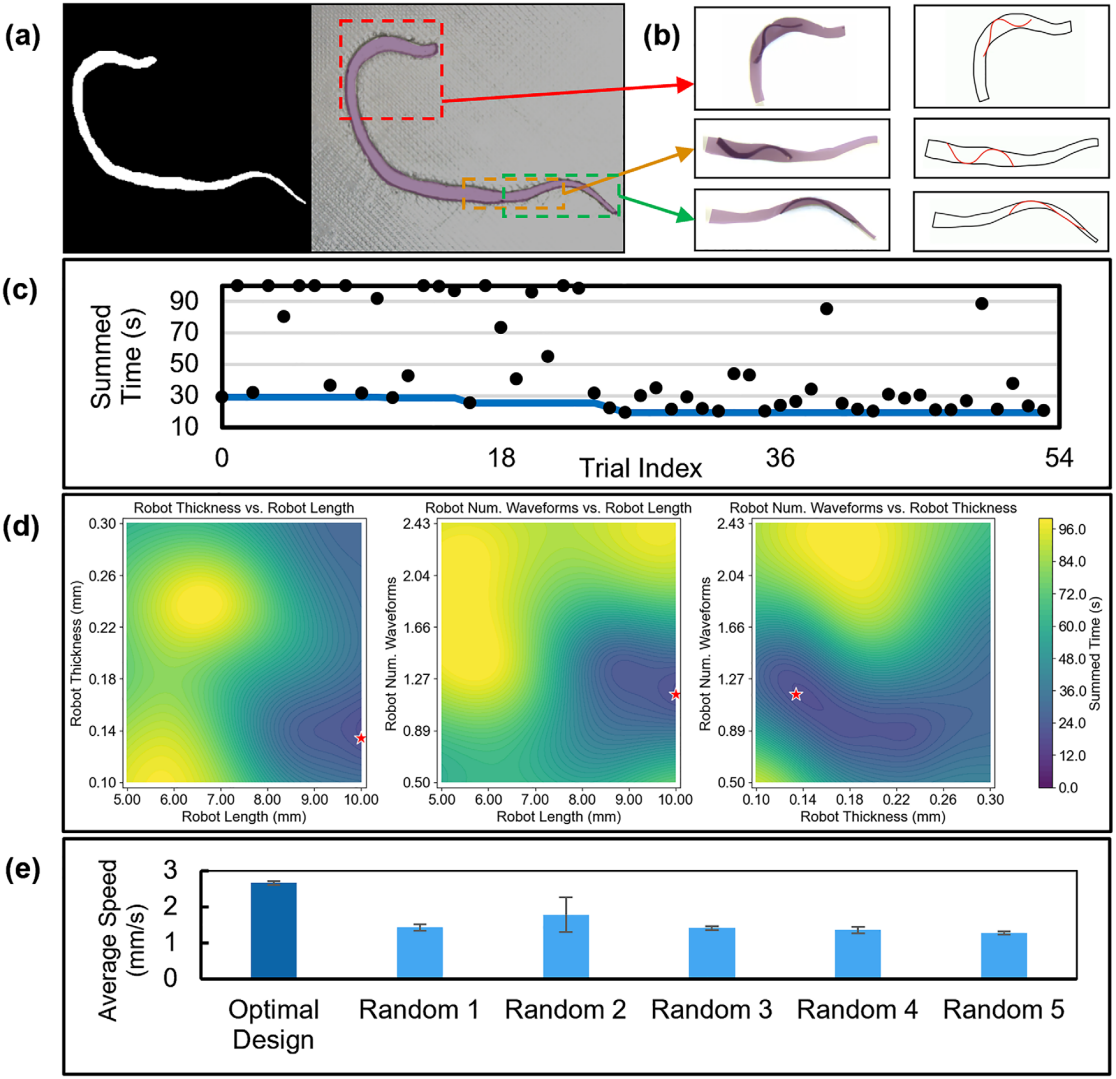
Bayesian optimization and experimental results for a right coronary artery‐mimicking channel. (a) Binary image of the extracted right coronary artery from an x‐ray angiogram (left), and the corresponding channel used for experimental validation (right). The channel was proportionally downscaled to a maximum width of approximately 2 mm. (b) Experimental snapshots (left column) and corresponding simulation results (right column) showing robot locomotion. Good agreement in locomotion and deformation is observed. The rows represent segments characterized by: (i) maximum width, midline curvature, and midline curvature change (top); (ii) maximum width change (middle); and (iii) minimum width (bottom). Colored arrows point to the corresponding segments in (a). (c) Optimization history from trial 0 to 53. Black dots represent individual trial results, while the blue line indicates the minimum identified summed time. Improvement plateaued at Trial 26. (d) Optimization landscapes for each design parameter pair. Darker purple regions correspond to lower total locomotion times. Red stars mark the identified optimal design parameter set. (e) Experimental comparison of robot speeds under the optimal design versus randomly generated ones (details of robots’ size can be found in Table S3). The optimal design exhibits the fastest speed. The error bar represents the standard variation (n = 6 total trials across N = 2 independent robots).

We further applied the segmentation method to a 2D channel mimicking the geometry of the right coronary artery, as illustrated in Figure 6a. The total locomotion time spent in each of the five segments was defined as the objective function. Since the computational burden was lower with the segmented approach, we allocated 24 trials to Sobol sampling and 30 trials to optimization. The optimization history is shown in Figure 6c, in which the lowest total time spent in segments decreased as the number of optimization trials increased. From trial 27 onwards, the results converged close to the optimum. Combined with the optimization contour plots in Figure 6d, these data show that the identified optimum was likely the global optimum. The optimized robot parameter set was identified as *L* = 10 mm, *t* = 0.13 mm, *w* = 1.16. Based on the average velocity measured along the entire track, the optimized robot achieved an average velocity of 2.66 ± 0.05 mm/s, significantly surpassing the velocities attained by five randomly selected sets of robot parameters (Figure 6e; Video S4). Furthermore, the optimized robot successfully avoided curling and getting stuck within the channel, further validating the effectiveness of the optimization algorithm.

## 7 Optimization for 3D Channel

We subsequently investigated whether the same design framework could be extended to channels mimicking 3D anatomical pathways. A coronary artery geometry was extracted from clinical imaging and downscaled for simulation (minimum width ≈ 0.5 mm, maximum width ≈ 2.3 mm). As with the previous case, the artery was partitioned into its five geometric bottleneck segments, and Bayesian optimization was performed using the total locomotion time across all segments as the objective function.

**FIGURE 7:**
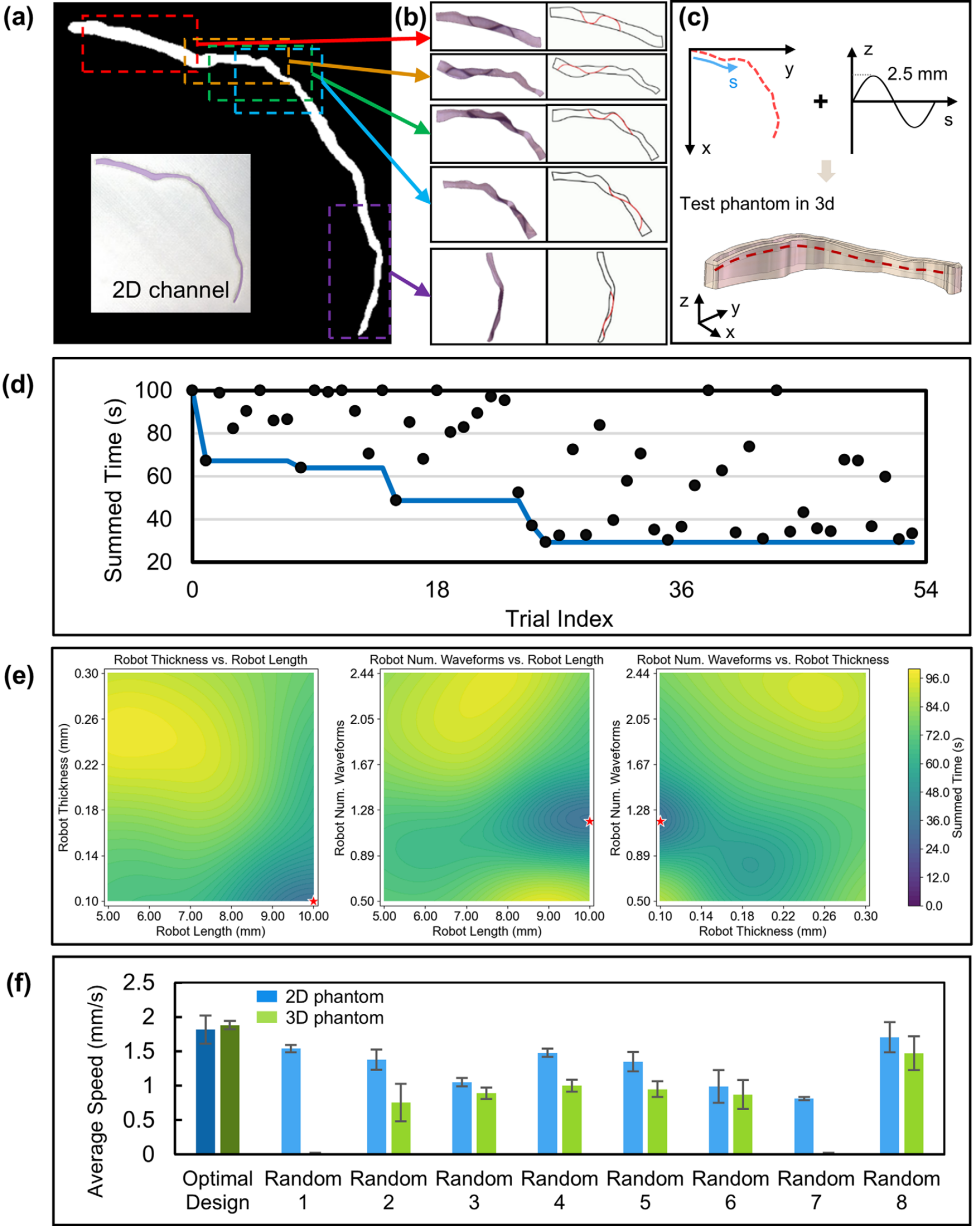
Bayesian optimization and experimental results for a left anterior descending artery ‐mimicking channel. (a) Binary image of the extracted left anterior descending artery from an x‐ray angiogram. The inset shows the channel used for experimental validation. The channel was proportionally downscaled to a maximum width of approximately 2 mm. (b) The left column shows experimental snapshots, while the right column displays corresponding simulation results of robot locomotion. Good agreement in locomotion and deformation is observed. From top to bottom, the rows represent segments characterized by: (i) maximum width, (ii) maximum midline curvature, (iii) maximum midline curvature change, (iv) maximum width change, and (v) minimum width. Colored arrows point to the corresponding segments in (a). (c) An out‐of‐plane undulation with an amplitude of 2.5 mm was applied to the 2D channel to transform it into a 3D channel. (d) Optimization history from trial 0 to 53. Black dots represent individual trial results, while the blue line indicates the minimum identified summed time. Improvement plateaued at Trial 26. (e) Optimization landscapes for each design parameter pair. Darker purple regions correspond to lower total locomotion times. Red stars mark the identified optimal design parameter set. (f) Experimental comparison of robot speeds under the optimal design versus randomly designed ones (details of robots’ size can be found in Table S4). The optimal design exhibits the highest speed in both 2D and 3D phantoms. The error bar represents the standard variation (n = 6 total trials across N = 2 independent robots).

Figure 7d shows the optimization history. Although the initial total locomotion time was farther from the optimum than in Figure 6c, the optimization converged to 29.2 s at trial 26, demonstrating a computationally efficient convergence. Combined with the optimization contours in Figure 7e, the converged design parameter set likely represents the global optimum. The optimal robot parameters were *L* = 10 mm, *t* = 0.1 mm, *w* = 1.18. This design achieved a higher average locomotion speed than multiple random baselines while avoiding coiling and jamming even at sharp bends. Importantly, this design was successfully transferred to a 3D locomotion scenario. When tested in an artery model with 2.5 mm out‐of‐plane undulation (Figure 7c), the robot maintained stable undulatory crawling and attained a comparable average speed, whereas some randomly selected designs stalled or became trapped (Figure 7f; Video S5).

Although 2.5 mm represents a relatively small out‐of‐plane variation, it is physiologically relevant. The human body contains millimeter‐scale anatomical tubular structures that exhibit locally quasi‐planar characteristics, with out‐of‐plane variations even smaller than this tested amplitude. For instance, geometric analyses of highly curved anatomical tubes, such as the internal carotid artery siphon [36] and semicircular canals [37, 38], demonstrate that their local trajectories are primarily defined by an osculating plane. Specifically, in semicircular canals, this non‐planarity is remarkably small, with a maximum deviation of only 1.5 mm induced by the ampulla [37, 38].

For 3D environments with larger out‐of‐plane variations, robots encounter not only the challenge of overcoming gravity when climbing steep slopes but also complex 3D contact mechanics. In such highly tortuous scenarios, purely planar undulatory gaits may struggle to maintain consistent wall contact and effective propulsion. Consequently, future 3D optimization frameworks must account for a more complex set of conditions, and the current channel segmentation strategy would need to be extended to identify and evaluate 3D‐specific bottlenecks, such as out‐of‐plane inclinations and spatial torsion.

## 8 Conclusions

In this work, we present a data‐efficient inverse‐design framework for magnetic soft millirobots navigating confined spaces. By coupling a Cosserat‐rod model with Bayesian optimization, the framework automatically identifies designs that achieve fast and reliable undulatory crawling without exhaustive simulations or large‐dataset‐training. Rather than a straightforward implementation of standard BO, we employed domain randomization that enables intrinsic robustness to environmental uncertainty for direct sim‐to‐real transfer without post‐fabrication tuning, while a segmentation strategy evaluates only the most critical channel bottlenecks, significantly reducing computational cost and improving the predictive accuracy of the surrogate model.

The optimized robots consistently outperformed arbitrarily selected baseline designs in both simulation and experiment. They exhibited stable locomotion without coiling, entrapping, or stalling across heterogeneous channel geometries and varying surface frictional properties, including coronary artery‐mimicking channels with out‐of‐plane undulations. These results demonstrate that the framework not only captures the nonlinear interplay between actuation, deformation, and contact mechanics but also provides a practical route toward task‐driven robot design in realistic environments.

While the proposed framework successfully navigates complex geometries by optimizing the most dominant design parameters, future iterations will address current modeling limitations to tackle more realistic anatomical environments. For instance, transitioning from 1D Cosserat rod approximations to fully 3D continuum mechanics models, incorporating hyperelastic constitutive laws, would allow us to capture intricate, large‐strain magneto‐elastic deformations much more accurately. Furthermore, the computational efficiency can be significantly improved by implementing more efficient collision detection algorithms, which will mitigate the heavy computational burden of exhaustive contact modeling. Crucially, dramatically accelerating the forward simulation will enable the optimization algorithm to process larger number of evaluations required to overcome the curse of dimensionality. Ultimately, this enhanced efficiency, alongside relaxing our assumptions of uniform magnetic fields, will allow the framework to expand its search space to higher dimensions, enabling the optimization of arbitrary continuous magnetization profiles and fully 3D boundaries, paving the way for highly tailored medical deployments.

## 9 Materials & Methods

For the fabrication of sheet‐shaped robots, a 1:1 mixture of PDMS and NdFeB was prepared and spread onto a glass plate using spacers to form a film. The film was then precisely laser cut into specific dimensions using a UV laser cutting machine (General Laser Tech.) to create the robots. Subsequently, the robots were rolled into a circular shape and magnetized in a 900‐mT uniform magnetic field. To minimize errors, their dimensions were measured using a handheld microscope (AM7915MZT, Dino‐Lite). Each experimental result was obtained by testing two different samples, with each sample tested three times (N = 2, n = 6), to reduce random experimental errors.

To evaluate fabrication reproducibility and experimental consistency, we tested three independently manufactured and magnetized samples with identical desired design parameters across two different channel widths (1.0 and 1.6 mm) (Figure S3). While One‐way ANOVA tests indicated minor statistical variations among the samples within the same channel (p = 0.0132 for the 1.0 mm channel; p = 0.0035 for the 1.6 mm channel), these maximum deviations (∼0.27 mm/s) were negligible compared to the highly significant performance shifts induced by variations in the environmental geometry (an absolute speed difference ranging from 1.8 to 2.3 mm/s, p = 2.27 × 10^−5^ when comparing the pooled sample data between the two channels). This statistical validation confirms that the locomotion trends are robust and that the fabrication‐induced noise does not compromise the reliability of our performance evaluations.

## Author Contributions

**Ziyu Ren** and **Wenqi Hu** proposed and designed the research. **Ziyu Ren** and **Chak Wang Tse** developed the algorithms. **Hong Wang**, **Yi Zheng**, and **Xi Chen** acquired experimental data. The simulation and experimental data were analyzed by **Ziyu Ren**, **Chak Wang Tse**, and **Hong Wang**. This research was supervised by **Ziyu Ren** and **Wenqi Hu**. All authors wrote the paper and participated in discussions.

## Conflicts of Interest

The authors declare no conflicts of interest.

## Supporting information




**Supporting File 1**: advs75714‐sup‐0001‐SuppMat.pdf.

**Supporting File 2**: advs75714‐sup‐0002‐VideoS1.mp4.

**Supporting File 3**: advs75714‐sup‐0003‐VideoS2.mp4.

**Supporting File 4**: advs75714‐sup‐0004‐VideoS3.mp4.

**Supporting File 5**: advs75714‐sup‐0005‐VideoS4.mp4.

**Supporting File 6**: advs75714‐sup‐0006‐VideoS5.mp4.

## Data Availability

The data that support the findings of this study are openly available in Github at https://github.com/tcwjac/Bayesian_Optimization_for_Sheet_Robots.
