# Supplementary material for: A Data‐Driven Inverse Design Methodology for Magnetic Soft Millirobots Navigating in Confined Spaces
Source: Adv Sci (Weinh). 2026 May 19:e75714. Online ahead of print. doi: 10.1002/advs.75714 (PMC13335899; doi:10.1002/advs.75714)
Supplement: Supplementary file 1 — Supporting File 1: advs75714‐sup‐0001‐SuppMat.pdf. [file ADVS-9999-e75714-s002.pdf]

Supplementary Materials for

A Data-driven Inverse Design Methodology for Magnetic Soft Millirobots Navigating in Confined Spaces

Ziyu Ren[†*a], Hong Wang[†b], Chak Wang Tse[†b], Yi Zheng[a], Xi Chen[b], Wenqi Hu[*b, c, d]

a School of Mechanical Engineering and Automation, Beihang University, Beijing, China.

b Department of Mechanical and Aerospace Engineering, The Hong Kong University of Science and Technology, Clear Water Bay, Kowloon, Hong Kong, China.

c Division of Integrative Systems and Design, The Hong Kong University of Science and Technology, Clear Water Bay, Kowloon, Hong Kong, China.

d Cheng Kar-Shun Robotics Institute, The Hong Kong University of Science and Technology, Clear Water Bay, Kowloon, Hong Kong, China.

* Corresponding authors. Email: wenqi@ust.hk, renzy@buaa.edu.cn.

† These authors contributed equally to this work as co–first authors.

The PDF file includes:

Note S1
Figs. S1 to S3
Table S1 to S4
Legends for videos S1 to S5

Supplementary Notes

Note S1: Identification of contact locations in numerical modeling

Accurate identification of contact locations is critical for evaluating the external contact force F_{cont} required to maintain penetration-free locomotion. Since the robot is mathematically represented by a 1D centerline in the Cosserat rod model, collisions are evaluated at discrete nodal locations rather than over continuous areas. The contact location identification algorithm is illustrated in Figure S1. Let Γ denote the discretized boundary points forming the channel walls. For a constituent computational node R_i on the robot's centerline, we compute the boundary segment vectors v and the relative position vectors w connecting the boundary points to R_i (Fig. S1a). A vector projection algorithm is implemented to project R_i onto the respective segments v, and the projection scalar is clamped to [0, 1] to restrict the contact point strictly within the bounds of each local segment (Fig. S1b). The closest valid projected point, which yields the minimum distance to R_i, is subsequently registered as the active contact location (Fig. S1c). This geometric evaluation is iterated across all robot nodes at each time step, robustly defining the active contact pairs, the local normal vectors n, and the penetration depth ϵ for force computations.

Supplementary Figures

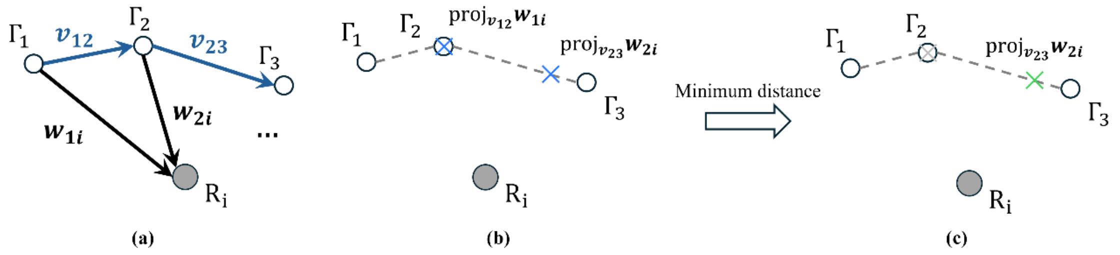

Fig. S1. Determination of robot-environment contact locations. (a) Geometric definition of boundary segment vectors v and relative position vectors w between the discrete boundary points Γ and a robot node R_i.(b) Orthogonal projection of w onto the respective boundary vectors v. The projected scalar is clamped between [0, 1] within the local coordinate frame of v, as demonstrated by $\mathrm{proj}_{v_{12}} w_{1i}$.(c) Evaluation of the Euclidean distances from the clamped projected points to R_i. The point yielding the minimum distance identifies the precise contact location.

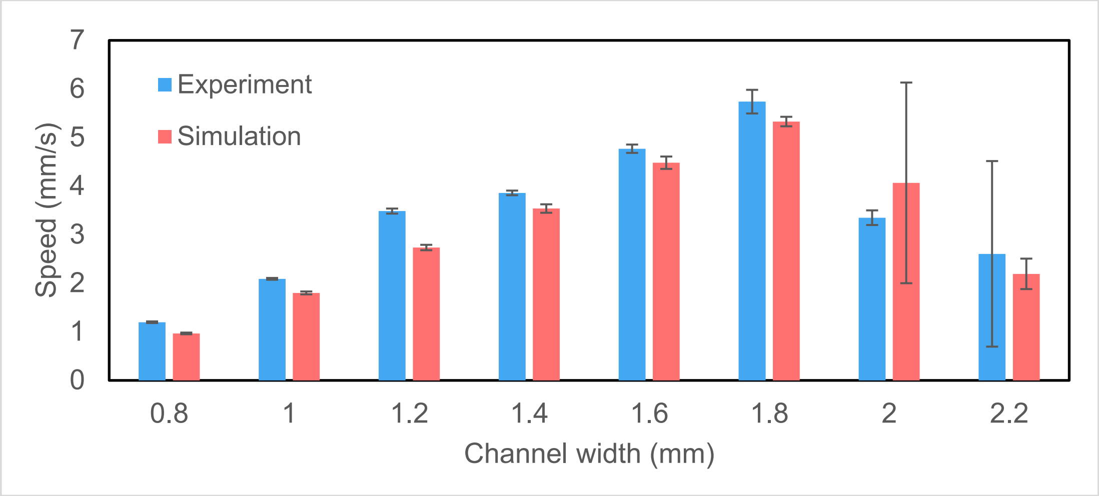

Fig. S2. Validation of fitted physics parameter set to a new robot. A new robot, measuring 9 mm in length, 3 mm in width, and 0.102 mm in thickness with a wavenumber w=1, was experimentally tested and simulated under identical actuation conditions and using the fitted physics parameter set from the manuscript. The simulated average speeds across various channel widths demonstrated strong agreement with the experimental average speeds in both magnitude and trend (Pearson correlation coefficient $r = 0.957$, $p < 0.001$). This strong correlation validates the fitted physics parameter set for simulating robots of different dimensions. The significant variation in speeds observed in the 2-mm and 2.2-mm channels is attributed to the robots' self-curving behavior, which was treated as a failure case with a speed of 0. Error bars represent the standard deviation ($n = 5$ for simulation and experiments).

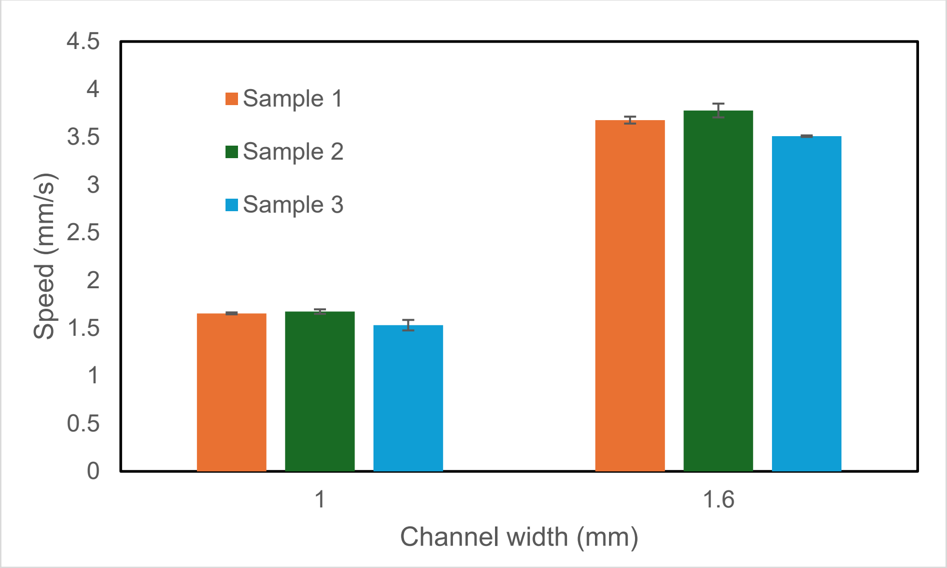

Fig. S3. Validation of fabrication reproducibility and experimental consistency. Three independently fabricated and magnetized samples with identical target parameters were tested across two channel widths (1.0 mm and 1.6 mm). While One-way ANOVA tests indicated minor statistical variations among the samples within the same channel ($p = 0.0132$ for the 1.0 mm channel; $p = 0.0035$ for the 1.6 mm channel), these maximum deviations (~0.27 mm/s) were negligible compared to the highly significant performance shifts induced by variations in the environmental geometry (an absolute speed difference ranging from 1.8 to 2.3 mm/s, $p = 2.27\times10^{-5}$ when comparing the pooled sample data between the two channels). This confirms that the macro-scale locomotion trends are robust and reproducible, and the fabrication-induced noise is negligible compared to the geometric effects of the confined environment. The error bar represents the standard variation (n=3, robot size: *L*, 9 mm, *b*, 3 mm, *t*, 0.16 mm, *w*, 1).

Supplementary Tables

Table S1 Random robots' design parameters for funnel-shaped channel

Robot Name	*L* (mm)	*t* (mm)	*w*	*b* (mm)
Optimal design	9.09	0.147	1.05	3
Random 1	14.35	0.215	0.90	3
Random 2	19.64	0.257	3.00	3
Random 3	15.65	0.181	1.50	3
Random 4	8.91	0.185	0.70	3
Random 5	9.00	0.176	0.90	3

Table S2 Robots' design parameters for serpentine channel

Robot Name	*L* (mm)	*t* (mm)	*w*	*b* (mm)
W/O Segmentation	9.38	0.18	0.82	3
With Segmentation	9.00	0.22	0.79	3
Random 1	9.80	0.201	0.70	3
Random 2	15.65	0.181	1.50	3
Random 3	8.91	0.185	0.70	3
Random 4	9.00	0.176	0.90	3
Random 5	9.09	0.147	1.00	3

Table S3 Robots' design parameters for right coronary artery-mimicking channel

Robot Name	*L* (mm)	*t* (mm)	*w*	*b* (mm)
Optimal Design	10.00	0.130	1.15	2
Random 1	6.30	0.225	0.65	2
Random 2	8.50	0.151	0.90	2
Random 3	7.00	0.170	0.80	2
Random 4	6.30	0.138	1.00	2

Random 5	10.00	0.140	1.70	2

Table S4 Robots' design parameters for left anterior descending artery-mimicking channel

**Robot Name**	***L*** **(mm)**	***t*** **(mm)**	***w***	***b*** **(mm)**
Optimal Design	10.00	0.100	1.18	2
Random 1	6.30	0.225	0.65	2
Random 2	9.80	0.156	1.00	2
Random 3	8.50	0.151	0.90	2
Random 4	7.00	0.170	0.80	2
Random 5	6.30	0.138	1.00	2
Random 6	10.00	0.140	1.70	2
Random 7	6.80	0.224	1.00	2
Random 8	10.00	0.130	1.15	2

Supplementary Videos

Video S1: This video demonstrates three typical failure cases of sheet-shaped robots crawling in confined spaces: coiling due to an overly wide channel, entrapment within an overly narrow channel, and stalling due to ineffective undulation.

Video S2: The first scene shows the robot crawling in a funnel-shaped channel under simulation. The second scene compares a robot with the optimal design against baseline designs in the funnel-shaped channel made of PLA. The third scene conducts a similar comparison in the funnel-shaped channel made of Mold Max 30.

Video S3: The first scene presents a simulation of the optimal design obtained through running optimization across the full channel. The second scene demonstrates simulations of the optimal design obtained via the segmentation strategy. The third scene compares the optimal and baseline designs in the serpentine channel.

Video S4: The first scene demonstrates simulations of the optimal design obtained by the segmentation strategy across various channel segments. The second scene compares the optimal and baseline designs in a coronary artery-mimicking channel.

Video S5: The first scene demonstrates simulations of the optimal design obtained by the segmentation strategy in various 2D channel segments. The second scene compares the optimal and baseline designs in a 3D coronary artery-mimicking channel.
